# Studying the Effect of Shortening Carbon Nanotubes via Ball Milling on Cellulose Acetate Nanocomposite Membranes for Desalination Applications

**DOI:** 10.3390/membranes12050474

**Published:** 2022-04-27

**Authors:** Nouran A. Elbadawi, Adham R. Ramadan, Amal M. K. Esawi

**Affiliations:** 1 Department of Chemistry, The American University in Cairo, New Cairo 11835, Egypt; nouran.ashraf@aucegypt.edu (N.A.E.); aramadan@aucegypt.edu (A.R.R.); 2 Department of Mechanical Engineering, The American University in Cairo, New Cairo 11835, Egypt

**Keywords:** carbon nanotubes, cellulose acetate, nanocomposite membranes, ball milling, desalination, nanofiltration

## Abstract

Studying the effect of different sizes of multi-walled carbon nanotubes (CNTs) on mixed matrix membranes in nanofiltration applications has not been widely reported in the literature. In this study, two different lengths of functionalized CNTs were used to investigate such effect. First, CNTs were shortened by using high-energy ball milling at 400 RPM, with a ball-to-powder weight ratio (BPR) of 120:1. Characterization of the structure of the CNTs was carried out using TEM, XRD, SEM, BET, and Raman Spectroscopy. Second, 0.001 wt % of unmilled and milled CNTs were incorporated into cellulose acetate nanocomposite membranes, Eli-0 (unmilled), and Eli-400 (milled at 400 RPM) to study their effects on the membranes’ morphology, porosity, hydrophilicity, and performance analysis in terms of permeation and salt retention rates of 5000 ppm Na_2_SO_4_. Results showed that shortening CNTs enhanced the membranes’ hydrophilicity and affected macrovoid and micropore formation. Furthermore, shortening CNTs resulted in opening their caps and improved the permeation rates with a slight adverse effect on salt retention.

## 1. Introduction

The availability of natural freshwater resources is decreasing primarily because of the rapid increase in population. The current population is expected to increase from 7.7 billion persons to more than 10 billion in less than 30 years [1]. An estimated 2.2 billion persons currently live without available clean and safe drinking water, according to UNESCO. Furthermore, it is expected that by the year 2050, more than 5 billion persons will face grave water scarcity, which would require the production of fresh water for drinking and irrigation from non-fresh water sources by desalination [2,3]. To date, the number of desalination plants has been estimated to be over 18,000, producing ~89 million cubic meters per day of fresh water, more than 50% of which is produced in the MENA region [4,5]. A multitude of desalination investigations have been conducted to optimize processes for obtaining cheap and efficient fresh water. Only a few technologies are considered viable enough for the large-scale production of fresh water, with membrane technologies such as Reverse Osmosis (RO), Micro, Ultra, and Nanofiltration taking the lead in the global market [6]. This is followed by thermal technologies, including Multi Stage Flash distillation (MSF) and Multi Effect distillation (MED) [7]. The energy requirements for thermal technologies in water desalination are generally higher than those of membrane technologies. For example, the total electrical energy consumption for MSF distillation is 13.5–25.5 kWh/m^3^, while that of RO is 5–9 kWh/m^3^ [8]. The high amount of energy required to separate water molecules from salt ions became a key parameter towards the upscaling of novel technologies on a commercial level [6,7]. The next generation of membrane desalination systems could therefore incorporate advanced nanocomposite membranes, offering simultaneously high permeation rates at the lowest operating pressures possible combined with high salt rejection capabilities.

The structure and properties of nanocomposite membranes depend on different variables such as the types of polymer matrices (e.g., polyamides, cellulose acetate), types of solvents (e.g., dimethylformamide, acetone), and the introduction of different additives, particularly nanofillers (e.g., graphene, nanoclays). Each variable can affect the porosity, surface, and cross section morphology of the membranes, thus creating mixed matrix membranes for different applications [9,10,11,12,13,14]. One of the most widely investigated types of nanofillers involves carbon nanotubes (CNTs), and investigations have aimed at understanding their effects on filtration performance, mechanical stability, and morphology changes. CNTs have been added in different amounts to different types of polymer matrices [15,16] and thin film composites [17,18] for a wide range of applications, including RO desalination [19], forward osmosis desalination [20,21], and contaminant filtration [22,23]. The general effects of CNTs on nanocomposite membranes is the change in morphology, porosity, and macrovoid formation, with potentially new channels available for solvent passage through the CNTs in addition to roughness and hydrophilicity changes. However, not enough studies have been carried out on the effect of different CNT sizes/surface areas on the morphology and performance of polymeric membranes in filtration applications. Usually, studies report that high aspect ratios of CNTs lead to superior properties in water treatment [10,24,25], however, very few have discussed how this works. Manawi et al. [26] fabricated polysulphone–CNT membranes with two different CNT aspect ratios (100–500 vs. 4000–20,000) in order to study the effect of the change of aspect ratio on porosity, flux, and mechanical properties. The small CNTs were functionalized by acid treatment, while the large ones were not. Pore sizes (analyzed with scanning electron microscopy (SEM)) and surface roughness (analyzed with atomic force microscopy) showed that the addition of both types of CNTs increased the porosity and roughness of the membranes as compared to the blank membranes, with the larger CNTs having a more pronounced effect. Furthermore, in a test for flux rates, the larger CNTs gave a better performance. Eslami et al. [27] studied the effect of the surface area of closed cap CNTs on NaCl and MgSO_4_ salt rejection in a gravity-driven permeation setup. Three different CNT shapes were grown by chemical vapor deposition on a Ni-coated porous Si support, giving different density and coverage values per surface area. The study showed that as the effective surface area of CNTs increased, salt rejection increased. The proposed reason was that the salt ions would get attracted to the surface of the CNTs instead of draining out with the permeate. As for the permeation rate, it was found that CNTs with a smaller surface area gave higher rates, but no explanation was offered for this. Trivedi et al. [28] studied the effect of different densities of vertically aligned CNTs in a poly-dimethylsiloxane composite on NaCl solution permeation and rejection rates. Having open caps, the CNTs acted as channels for water permeation, with the highest density exhibiting the highest permeation rate. The study also found that salt rejection rates were independent of the densities, and that they depended mainly on the charges on the surface of the polymeric matrix. Other investigations have studied the effect of CNTs’ aspect ratio on different composite materials for mechanical applications. These studies typically incorporated from two to three ratios of CNTs, and the membranes were prepared by melt mixing or curing. The results were focused on the effects on the electrical, mechanical, rheology, and thermal properties of the composites [29,30]. The common observation was that the addition of CNTs changed the properties of the composites, however, each matrix behaved differently with different preparation conditions and CNT types [29,30,31,32]. Detailed experimental analyses on the role of different sizes of CNTs on polymer-based nanocomposite membranes have not been widely conducted. In this respect, the current study focuses on the effect of varying CNT sizes on membrane morphology, porosity, and hydrophilicity, as well as on membrane performance in terms of permeation and salt rejection rates in nanofiltration applications.

## 2. Materials and Methods

Elicarb multi-walled carbon nanotubes with an average diameter of 10–12 nm and length > 1 µm (Thomas Swan, Consett, UK) were functionalized, shortened, and used as nanofillers. Cellulose Acetate (CA), with an average molecular weight of 50,000 Da and 39.7 wt % acetyl content (Aldrich, St. Louis, MO, USA), was used as the polymer matrix. Acetone (Merck KGaA, Darmstadt, Germany, purity ≥ 99.5%) was used as solvent, and deionized water as non-solvent. Sodium sulfate (Sigma Aldrich, St. Louis, MO, USA, purity ≥ 99%) was used for salt retention determinations.

### 2.1. CNT Functionalization, Shortening, and Characterization

Prior to shortening, the CNTs were functionalized as follows: 3 g of CNTs were placed in a round flask, in 100 mL of a mixture of concentrated acids (H_2_SO_4_:HNO_3_ = 3:1). The mixture was sonicated in an ultrasonic bath at less than 15 °C for 30 min, and then the mixture was refluxed at 90 °C raised to 130 °C over a period of 100 min. The solution was later cooled down to room temperature at 21 °C, and then the CNTs were filtered off using Whatman PTFE filter membrane (0.25 µm) and washed to neutral pH [33]. The success of the functionalization was verified using Nicolet 380 FTIR with the KBr pellet method, where peaks indicating the presence of carboxylic groups were detected.

For shortening the CNTs, a planetary ball mill (PM400, Retsch, Haan, Germany) was used. Milling was conducted using 6 mm diameter stainless steel balls. The ball-to-powder ratio (BPR) was 120:1, and the process was carried out in 125 mL stainless steel jars [34]. Total process time was 1.5 h, with increments of 15 min on and 10 min off to avoid the overheating of the samples; milling speed was set to 400 RPM to effectively produce short-length CNTs.

Characterization of the CNTs was conducted using TEM (JEM-2100, JEOL, Tokyo, Japan) and SEM (Leo Supra 55, Zeiss, Oberkochen, Germany). Sample solutions were prepared in the following manner. About 0.0001 g of CNTs were weighed and put in 50 mL isopropanol. The samples were then sonicated for 15 min at less than 10 °C. For the TEM analysis, the copper grid was suspended in the middle section of the suspension solutions to minimize the amount of agglomerates attached to the grid. As for the SEM, single drops of about 0.1 mL from the middle section of the solution were placed on copper substrates and dried at 50 °C for 5 min. This facilitated the imaging of individual nanotubes. ImageJ software was used to determine the length distributions of the CNT samples. Statistical analysis was carried out for 200 individual nanotubes to check for outliers and length distribution [35]. X-ray diffraction (XRD) was carried out using a D8 Bruker x-ray powder diffractometer at 40 kV and 30 mA using CuKα (λ = 0.1542 nm). BET surface areas were characterized using the N_2_ adsorption method at 77 K (ASAP 2020, Micromeritics, Norcross, GA, USA), while Raman spectroscopy (Enwave Optronics, Irvine, CA, USA) was used at 785 nm to analyze the change in the bonding properties of the nanotubes. Finally, the Boehm acid base back-titration, the procedure for which is detailed elsewhere [36], was used to evaluate the carboxylic groups found on the nanotube surface using Na_2_CO_3_ as base and HCl as acid.

### 2.2. Membrane Preparation and Characterization

**Figure 1:**
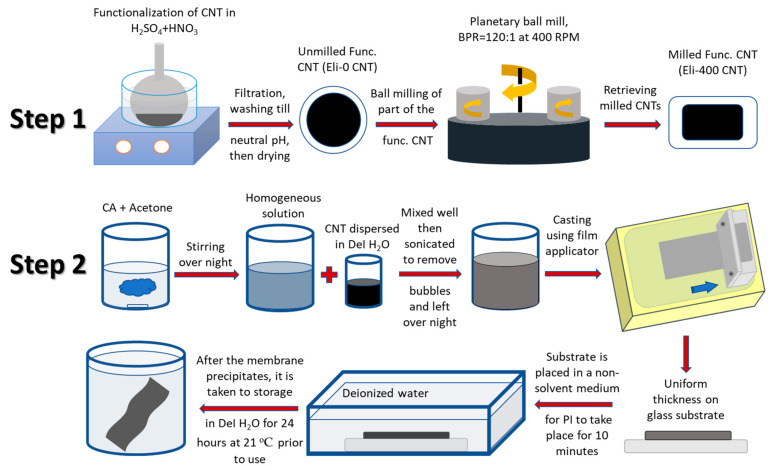
Schematic diagram of the preparation procedure of nanocomposite membranes.

Nanocomposite membranes using CA as a polymer matrix and CNTs as nanofillers were fabricated using wet chemistry. The composition of the cast solution was 15% CA, 0.001% CNTs, and 19% deionized water (DeI water). Acetone was the solvent. Membranes were cast to have a final thickness of around 97 ± 3 µm, and phase inversion (PI) was used to produce the membranes. All samples were prepared in triplicates [37]. Figure 1 shows a schematic diagram of the preparation procedure.

Characterization was conducted as follows: SEM was used to evaluate the morphology of the membranes, along with their final thickness. FTIR using the KBr method was used to identify the type of interaction between the CNTs and the cellulose acetate polymer matrix. Porosity analysis using pore size distribution and BET surface areas was performed using N_2_ adsorption at 77 K with an ASAP 2020-Micromeretics apparatus. Hydrophilicity analysis was carried out using the sensile drop method to measure the contact angle (Krüss GmbH, Hamburg, Germany) between a drop of distilled water and the surface of a dry, uniformly positioned membrane, and an average of three measurements were taken.

### 2.3. Separation Analysis

Permeation tests were carried out at 21 °C room temperature, for the samples using a 5000 ppm Na_2_SO_4_ salt solution, in a dead-end cell (HP4750 stirred cell, Sterlitech, Auburn, WA, USA) with an active surface area of 14.6 cm^2^. This salt was selected because it gives higher salt retention rates in comparison to NaCl and MgSO_4_ [17,38]. The membranes were first subjected to high pressure at 26 bars for 30 min prior to testing, using deionized water to eliminate the compression effect on the samples. The pressure was then reduced to 15 bars, and the permeation rate was evaluated using Equation (1).

(1) $$J=\frac{V}{A.t.P}$$

where *J* (in L/m^2^h·bar) is the permeation rate, *V* (in L) is the permeate volume, *A* (in m^2^) is the active area of the membrane in which separation takes place, *t* (in h) is the time it takes for the permeate to produce the volume *V*, and *P* (in bar) is the applied pressure. Salt retention was evaluated using the conductivity meter (Jenway, Staffordshire, UK) and calibration curve methods. The concentration of the permeate was calculated in ppm, and then using Equation (2), the retention percentage was calculated:

(2) $$R=100\times \left(1-\frac{C_{p}}{C_{f}}\right)$$

where *R* (%) is the salt retention percentage, *C_p_* (in ppm) is the salt concentration of the permeate, *C_f_* (in ppm) is the salt concentration in the feedwater. All measurements were performed in triplicates for each membrane type.

## 3. Results

### 3.1. Characterization of Shortened CNTs

**Figure 2:**
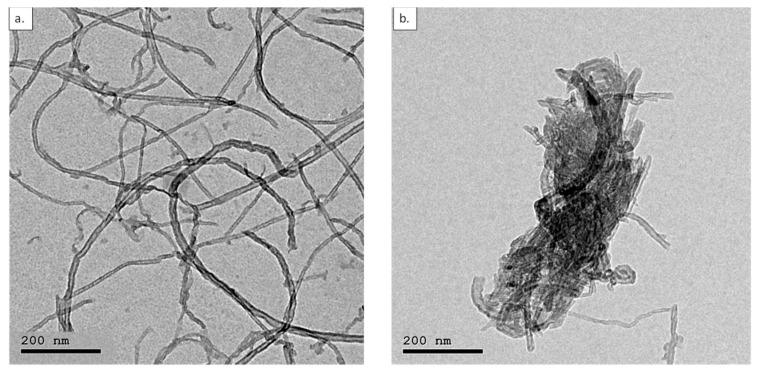
TEM images of (**a**) Eli-0 and (**b**) Eli-400 CNTs at 200 nm scale.

TEM images of CNTs, Figure 2, show the functionalized unmilled Elicarb CNTs (Eli-0) as well as the same CNTs after ball milling at 400 RPM for 1.5 h (Eli-400).

**Figure 3:**
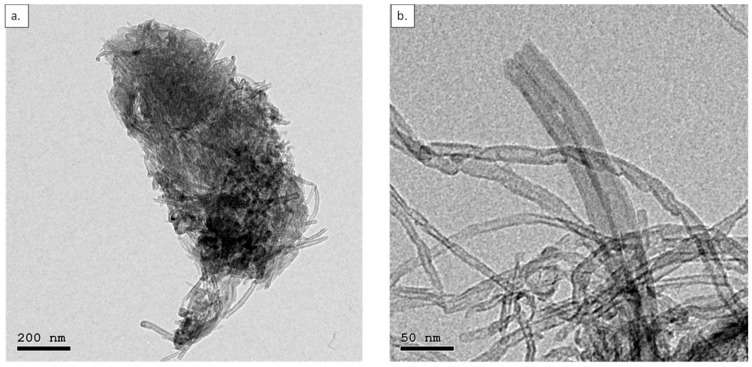

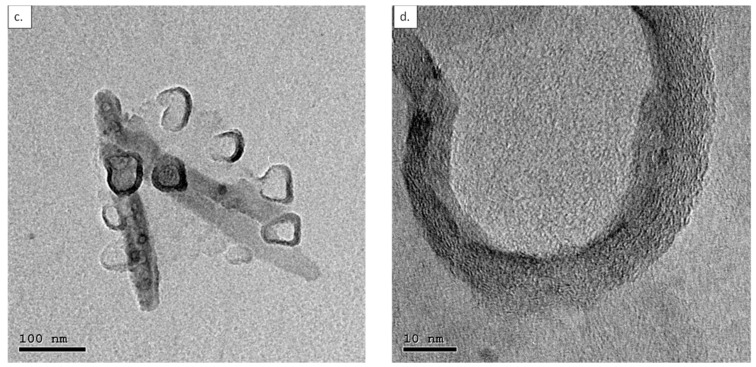
TEM of Eli-400 CNTs: (**a**) CNT agglomerate, (**b**) magnified part of the agglomerate to show the open cap, (**c**) damaged CNTs, and (**d**) magnified part of the damaged CNTs showing the graphene walls.

TEM images of Eli-0 and Eli-400 clearly show how ball milling was able to shorten and open the caps of the CNTs. Imaging Eli-400 was challenging because the shortened/damaged CNTs tended to cluster into small bulks. Upon further investigation, it was observed that in addition to cutting the nanotubes into shorter lengths, ball milling caused the partial damage of the CNTs, transforming them into graphene-like sheets, as shown in Figure 3.

Figure 3a shows a cluster of shortened CNTs. Figure 3b is a representation of how ball milling managed to open some of the caps of the CNTs. Figure 3c,d show the damaging effect of the high-speed 400 RPM ball milling on the CNTs, where Figure 3d is a zoomed view of the multi-walled graphene-like sheets present in the circular conformation shown in Figure 3c.

**Figure 4:**
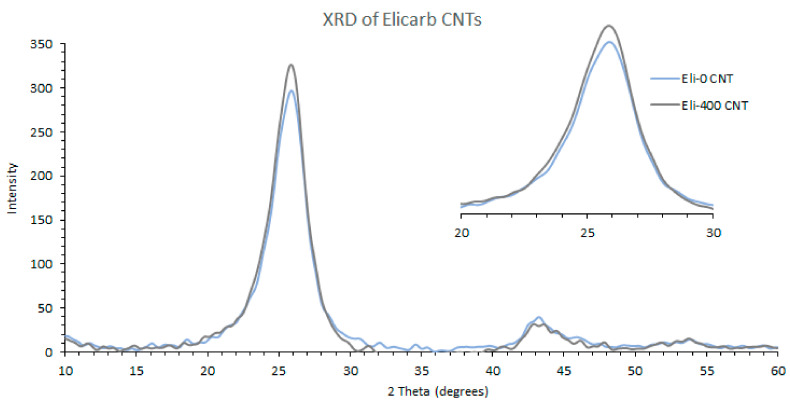
XRD spectrums of unmilled and milled Elicarb CNTs.

To give a better understanding of the effect of ball milling on the structure of the CNTs, results of the XRD analysis of Elicarb CNTs before and after shortening are displayed in Figure 4.

The three main peaks of CNTs are shown in Figure 4. The first peak at 26° corresponds to the (002) inter-planar spacing of 0.34 nm between the graphene sheets of the multi-wall Elicarb CNTs, while the second and third peaks at 43° and 54° correspond to the (100) and the (400) planes, with *d* spacings equal to 0.21 and 0.17 nm, respectively [39]. Figure 4 also has a zoomed view of the (002) peak to show the effect of ball milling on the structure of the nanotubes, where the peak became only slightly broadened. The XRD analysis thus indicates that the majority of the CNTs maintained their tubular structure, which agrees with reports in the literature [40].

**Figure 5:**
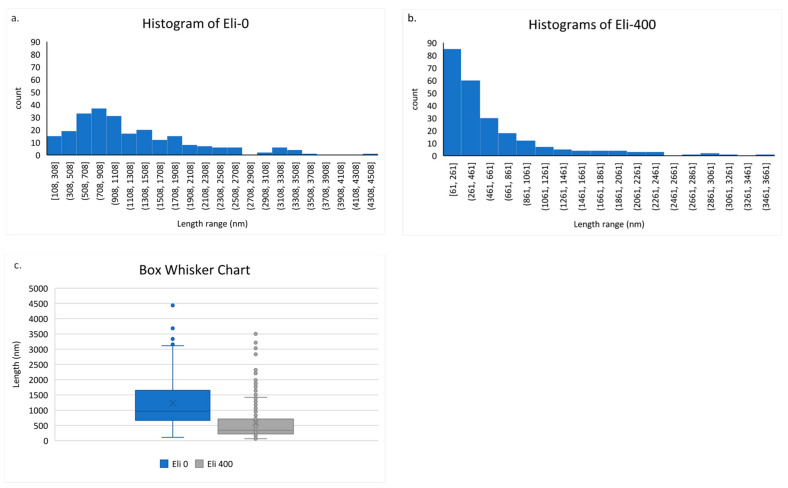
(**a**) Histogram of length distribution of Eli-0 CNTs, (**b**) histogram of length distribution of Eli-400 CNTs, and (**c**) Box Whisker chart of Eli-0 and Eli-400 CNTs.

Length measurements from the SEM images were carried out using ImageJ software and were tabulated. The histogram results and the Box Whisker chart are shown in Figure 5.

Figure 5 gives a clear idea about the effect of using ball milling in shortening CNTs. Figure 5a shows a wide distribution of lengths of unmilled Elicarb, while Figure 5b shows the narrowing of the range of length distribution and a shift to lower values. Figure 5c supports the histogram analysis of the narrowing down of the length distribution due to ball milling.

Statistical analysis using quartile calculations on Excel was performed to give an estimate of the lengths’ population distribution, identify the outliers, and the mean values. Table 1 shows the size distribution.

According to Table 1, 75% of the total nanotubes’ population lengths decreased by an amount of ~56% for Eli-400, while their means decreased by ~61%. As for the BET SA characterization technique, the results show that the surface area of the nanotubes increased after ball milling, indicating more surface exposure due to the shortening and the opening of the caps of the nanotubes.

**Figure 6:**
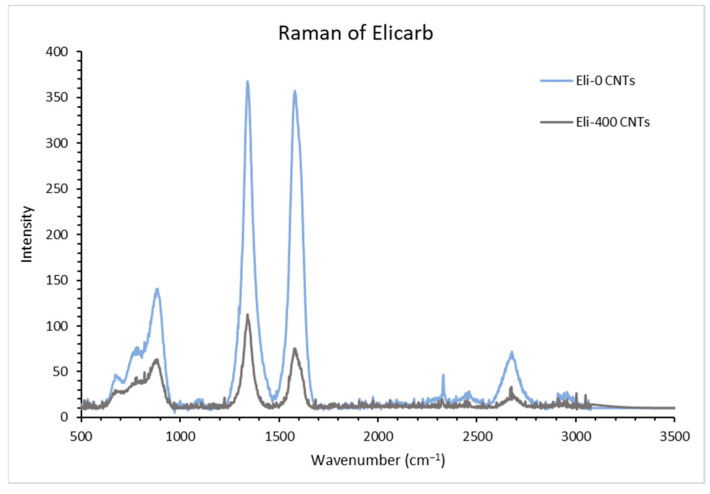
Raman spectra of the milled and unmilled Elicarb CNTs.

Raman spectroscopy was used to evaluate the amount of damage that took place on the surface of the CNTs due to the milling process. Important bands to focus on include the G (graphite) band (~1585 cm^−1^), commonly found in any carbon-based material with sp^2^ bonding due to the in-plane stretching mode, and the D (defect) band (~1350 cm^−1^), found when the sp^2^ bonding in the hexagonal structure exhibits any defects. Figure 6 shows the Raman spectra of the milled and unmilled Elicarb CNTs [41].

Based on the Raman spectra of the milled and unmilled Elicarb CNTs in Figure 6, it is clear that the band intensities decreased due to the damage that took place in the structure of the graphene walls. An interesting feature of the curves was found at about ~880 cm^−1^. This is suggested to be due to an armchair chirality conformation, associated with semiconductor type nanotubes [42]. The ratio of the intensity of the D and G bands (I_D_/I_G_) is usually used as a further indication and verification of the amount of damage of the graphene structure. In Table 2, the recorded values are presented.

As shown in Table 2, the ratio increased as more damage took place because the intensity of the D bands increases with the introduction of more defects, while the intensity of the G band decreases due to the presence of a less ordered structure [40,43]. The Raman results are in agreement with the TEM results.

An additional characterization technique using Boehm back-titration was performed to evaluate the carboxylic groups found on the surface of the CNTs. Titration performed for Eli-0 and Eli-400 gave approximately the same amount of ~0.2 mmol/g for the carboxylic groups. This indicates that ball milling did not affect the amount of acidic sites on the nanotubes.

### 3.2. Characterization of the Nanocomposite Membranes

**Figure 7:**
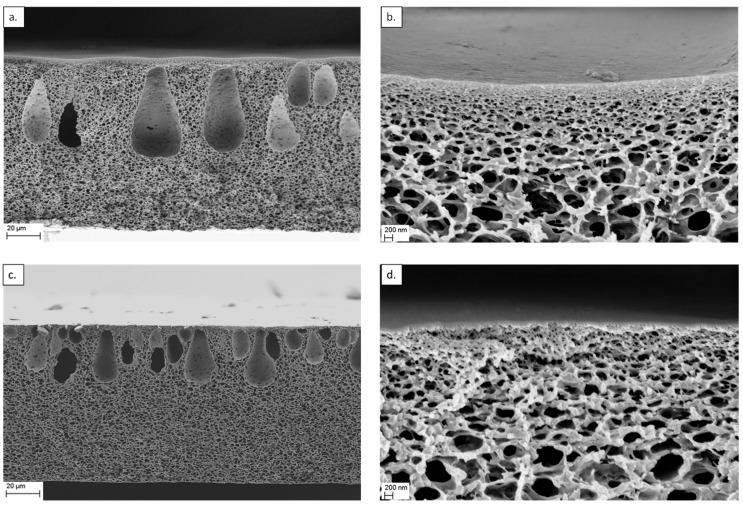
SEM of the cross-section of CA nanocomposites with 0.001% (**a**,**b**) Eli-0 and (**c**,**d**) Eli-400 CNTs at two different magnifications.

Characterization of the cross-sectional area of the membranes was conducted by SEM. Sample images are presented in Figure 7. Eli-0 membranes have several classical drop-like macrovoids which extend across the cross section of the membranes. It is clear that shortening the CNTs by mechanical milling has an effect on the membrane pore structure. The morphology of the Eli-400 membranes shows smaller macrovoids which are larger in quantity and are positioned closer to the dense layer of the membrane.

The phase inversion process entails the demixing of the solvent/non-solvent when a cast solution is introduced into the non-solvent bath. The speed at which this process takes place plays a major role in determining the morphology of the cross section of the precipitated membranes, as well as the shapes and sizes of the formed macrovoids [44]. Figure 7a,c show two different shapes of macrovoid structures. Two factors could have contributed to this difference. The first one was the change in viscosity of the casting solution, expected to slow the rate of the demixing process during PI. This was possibly due to the increase in the quantity of the fragments of the carbon nanotubes at the same weight percentage. This promoted better interaction between the polymeric chains on the one hand, and increased the viscosity of the solution on the other, thus preventing macrovoids from expanding in size [45,46]. The second factor had an opposite effect, where the short Eli-400 nanotubes would have a wider dispersion in the membrane cast solution, creating more hydrophilic sites and promoting the demixing of the solvent-non-solvent in the phase inversion step [47]. Consequently, the macrovoids increased in number, but not in size.

**Figure 8:**
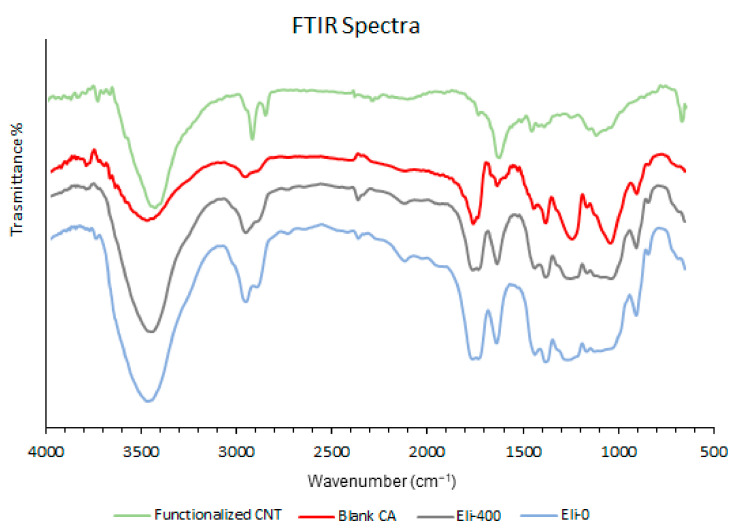
FTIR spectra of functionalized carbon nanotubes, powder cellulose acetate, Eli-0 membrane, and Eli-400 membrane.

Figure 8 shows the FTIR spectra of Eli-0 and Eli-400 nanocomposite membranes in comparison to the spectra of blank cellulose acetate powder and the spectra of functionalized CNTs. The main absorption peaks for the blank cellulose acetate powder were identified at 3460 cm^−1^ (O-H bonds), 2945 cm^−1^ (C-H bonds), 1757 cm^−1^ (C=O bonds), 1632 cm^−1^ (C-C bonds), 1379 cm^−1^ (CH_2_ bonds), 1230 cm^−1^ (C-O bond), 1032 cm^−1^ (C-O-C bonds), and 899 cm^−1^ (C-H bonds) [48]. As for the main absorption peaks for the functionalized CNTs, they were found at 3425 cm^−1^ (O-H bonds), 2921 and 2850 cm^−1^ (C-H bonds), 1737 cm^−1^ (C-O bonds), 1625 cm^−1^ (C-C bonds), 1454 and 1391 cm^−1^ (CH_2_/CH bonds), 1245 cm^−1^ (O-H bonds), and 1116 cm^−1^ (C-O bonds) [49].

On comparing the FTIR spectra of both Eli-0 and Eli-400 membranes with the peaks of the blank cellulose acetate powder in Figure 8, it is clear that the location of the main absorption peaks did not show any significant shift, and no new peaks appeared, thus confirming that the type of interaction between the CNTs and the cellulose acetate polymer matrix is a physical interaction (via hydrogen bonding) and not a chemical one. However, the shape of a few peaks changed, particularly down the wavenumber, where they became broader. This is probably because the functionalized CNTs’ absorption overlapped with that of the cellulose acetate in the same regions, making the peaks stronger and broader.

**Figure 9:**
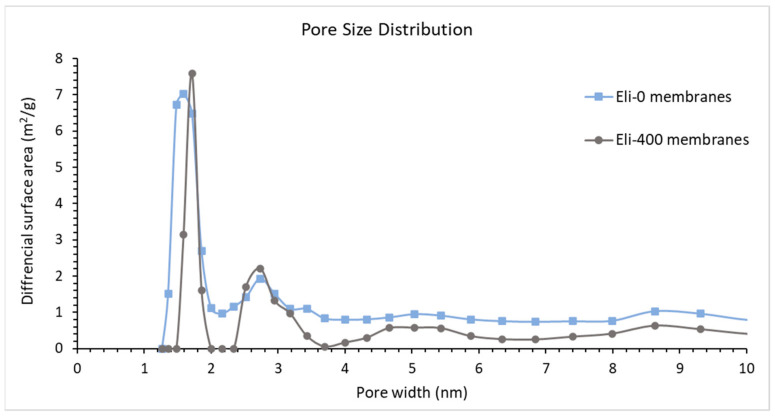
Differential pore areas versus pore width for the two nanocomposite membranes.

Figure 9 shows the pore size distribution of the nanocomposite membranes in terms of the differential surface areas (m^2^/g) versus pore width (nm), analyzed using the Direct Density Function method for the pores that were smaller than 10 nm.

Pores that are smaller than 5 nm are believed to play important roles in the salt rejection of membranes, as highlighted in our previous work [37]. Eli-400 membranes have a high pore surface area in this region (~1.7 and ~2.7 nm), while Eli-0 membranes have high surface areas for smaller pores (~1.3 and 1.5 nm) and slightly less surface area for pores at ~2.9 nm. This could explain the dense skin structure in Figure 7b,d, where Eli-0 membranes have a denser top layer with smaller pores compared to the Eli-400 membranes. The BET surface area data for the membranes are displayed in Table 3, along with the contact angle measurements.

The results show that the Eli-400 membranes have a higher BET surface area, which agrees with the finding of the pore size distribution in Figure 9, as well as the fact that Eli-400 CNTs have open caps, as shown previously in Figure 3b. As for the contact angle measurement, the range is narrow, showing that Eli-400 membranes exhibit higher hydrophilicity than Eli-0 membranes. This is possibly due to the better dispersion of the shorter CNTs.

### 3.3. Permeation and Salt Retention Rates

The results of the performance of the nanocomposite membranes in terms of permeation and salt retention rates, for a 5000 ppm Na_2_SO_4_ salt solution at 15 bars, are displayed in Table 4. Furthermore, a collection of all the data above is displayed in the same table to facilitate the comparison and analysis in the discussion section.

Table 4 shows that the permeation rate increased by ~63% for the short open-capped Eli-400 CNT composite membranes compared to the long closed-capped Eli-0 CNT membranes. This had a slight adverse effect on the salt retention rate, which decreased by only 1%.

## 4. Discussion

The focus of this work was to investigate how the shortening of CNTs using ball milling affects the morphology, porosity, and performance of nanocomposite membranes. The first clear correlation observed was between the BET surface area of the CNTs and the morphology change of the membranes, where a larger surface area of the shortened CNTs was found to indicate more CNTs per unit weight, thus creating more interaction at the CNT–CA polymer interface. Such interactions changed the shape and size of the macrovoids, as explained earlier, as well as the porosity of the dense skin layer. That would also explain the differential pore size distribution values, where Eli-0 membranes showed a high differential surface area, and therefore quantity, for the smaller 1.3 and 1.5 nm micropores, while Eli-400 membranes had a high surface area for the larger 1.7 nm micropores. The second correlation found was between the CNT length and the hydrophilicity of the membranes; the shorter the CNTs, the more hydrophilic the membranes. Although the difference between the two data values is not big, it is still detectable. Enhanced hydrophilicity could possibly be due to the increase in the quantity of the nanotubes per weight, which then allows more carboxylic sites to become available and consequently increases hydrophilicity.

The observed change in membrane morphology was reflected in the performance of the membranes. The permeation rate increased with an increase in the BET surface area of the membranes. CNT morphology could have contributed to this finding in terms of the open caps of the Eli-400. This is expected to allow permeation rates to increase through the channels that became available. This may have also contributed to the increase of the BET surface area of the corresponding membranes in comparison to closed-capped Eli-0 CNT membranes. The increased permeation of Eli-400 membranes was coupled with a minimal change in salt retention performance. This could be explained in light of the comparable pore surface area of the two membranes for pores smaller than 3 nm, and the lower pore surface area for the Eli-400 membrane for pores beyond 3 nm. This is also corroborated by the SEM images of Figure 7, where Eli-0 membranes have a denser top layer with smaller pores compared to the Eli-400 membranes.

## 5. Conclusions

The same concentration of CNTs of two different lengths was found to affect the polymer matrix morphology, porosity, and accordingly, membrane performance. Shorter CNTs were found to significantly enhance the flux with only a minimum impact on salt rejection. Further ongoing work includes investigating the use of lower ball milling energies to preserve the structure of the CNTs while shortening them, and conducting a more comprehensive comparison between commercial CNTs with different aspect ratios and ball-milled CNTs in order to investigate the effect of the size/surface area/aspect ratio of CNTs on the morphology, porosity, and performance of nanocomposite membranes. This might open up a potential for different applications, including micro and ultrafiltration as well as waste water treatment.

## Figures and Tables

**Table 1:** Length distribution (nm) and BET surface area (SA) (m^2^/g) after ball milling of functionalized Elicarb CNTs.

	Quartile 1 (25% of Population)	Quartile 3 (75% of the Total Population)	Quartile 2 (Median)	Mean Excluding Outliers	BET SA (m^2^/g)
**Eli-0 CNTs**	666	1649	965	1132	79
**Eli-400 CNTs**	223	710	342	421	113

**Table 2:** Summarized results of Raman shifting.

	D	I_D_	G	I_G_	I_D_/I_G_
**Eli-0 CNTs**	1340	368.0	1580	357.5	1.03
**Eli-400 CNTs**	1340	112.8	1584	73.9	1.53

**Table 3:** BET surface area and contact angle of the membranes.

	BET SA of Membranes (m^2^/g)	Contact Angle
**Eli-0 membranes**	5.8 ± 1.3	64 ± 4
**Eli-400 membranes**	7.2 ± 0.3	61 ± 1

**Table 4:** Summary of all data.

	CNTs Length (nm)	CNT Diameter (nm)	CNT BET SA (m^2^/g)	Membranes BET SA (m^2^/g)	Membranes Contact Angle	J Na_2_SO_4_ (L/m^2^h·bar)	Salt Retention %
**Eli-0**	1132 ± 661	11 ± 1	79	5.8 ± 1.3	64 ± 4	0.41 ± 0.01	97.5 ± 0.3
**Eli-400**	421 ± 299	11 ± 1	113	7.2 ± 0.3	61 ± 1	0.67 ± 0.02	96.5 ± 0.9

## Data Availability

Not applicable.
